# Supplementary figures and images for: Whole genome sequencing to study SARS-CoV-2 transmission between university students and the surrounding community in Pittsburgh, Pennsylvania, 2020–2021
Source: Antimicrob Steward Healthc Epidemiol. 2026 Mar 23;6(1):e62. doi: 10.1017/ash.2026.10307 (PMC13104529; doi:10.1017/ash.2026.10307)

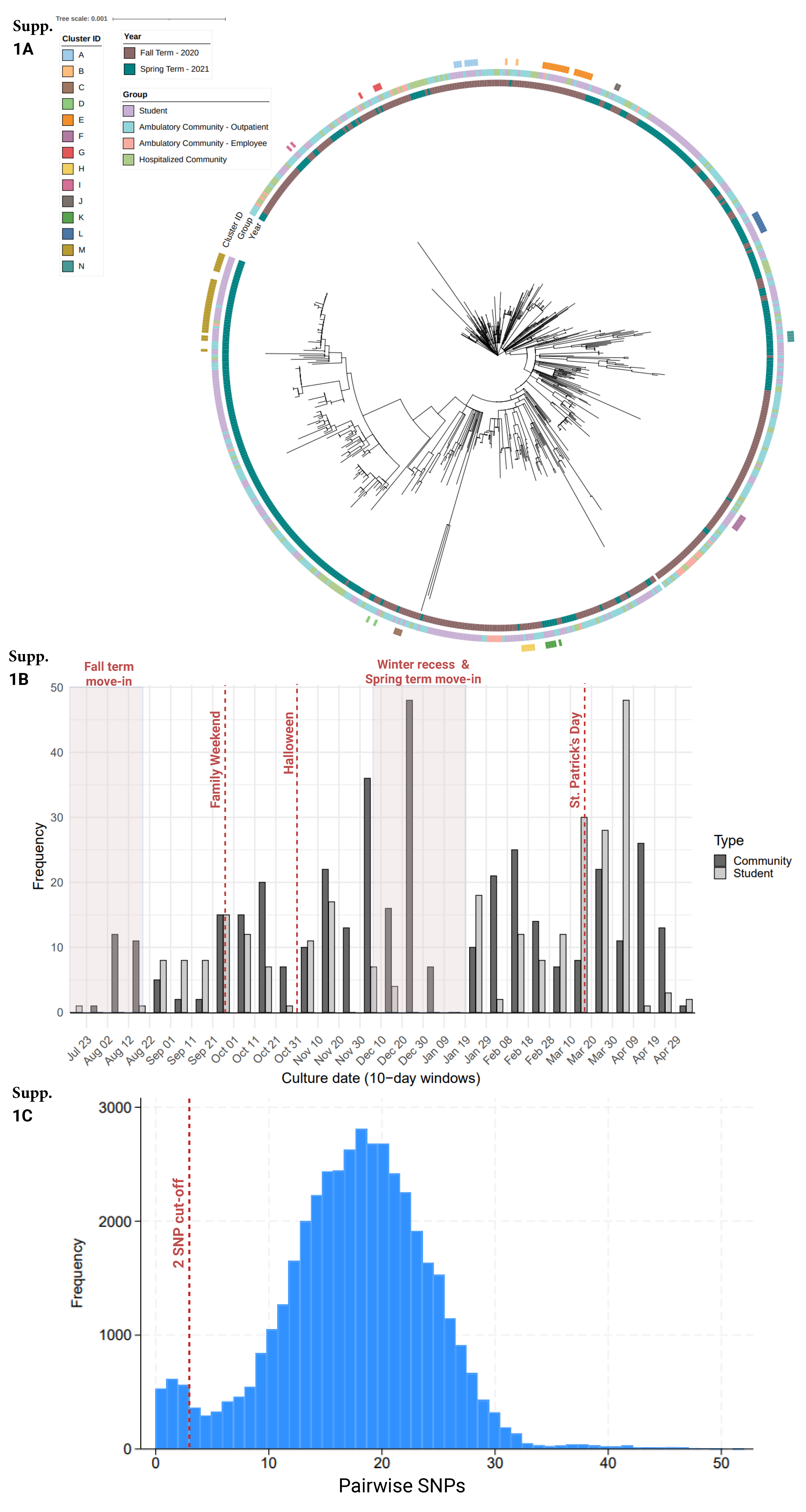

Supplement: Rangachar Srinivasa et al. supplementary material 1 — Rangachar Srinivasa et al. supplementary material [file S2732494X26103076sup001.tiff]
